# Genetics, sleep and memory: a recall-by-genotype study of *ZNF804A* variants and sleep neurophysiology

**DOI:** 10.1186/s12881-015-0244-4

**Published:** 2015-10-24

**Authors:** Charlotte Hellmich, Claire Durant, Matthew W. Jones, Nicholas J. Timpson, Ullrich Bartsch, Laura J. Corbin

**Affiliations:** School of Physiology and Pharmacology, University of Bristol, Bristol, UK; Clinical Research and Imaging Centre (CRICBristol), University of Bristol, Bristol, UK; MRC Integrative Epidemiology Unit at University of Bristol, Bristol, UK

**Keywords:** Sleep, Memory, Schizophrenia, Spindles, *ZNF804A*, rs1344706, Recall-by-genotype, ALSPAC

## Abstract

**Background:**

Schizophrenia is a complex, polygenic disorder for which over 100 genetic variants have been identified that correlate with diagnosis. However, the biological mechanisms underpinning the different symptom clusters remain undefined. The rs1344706 single nucleotide polymorphism within *ZNF804A* was among the first genetic variants found to be associated with schizophrenia. Previously, neuroimaging and cognitive studies have revealed several associations between rs1344706 and brain structure and function. The aim of this study is to use a recall-by-genotype (RBG) design to investigate the biological basis for the association of *ZNF804A* variants with schizophrenia. A RBG study, implemented in a population cohort, will be used to evaluate the impact of genetic variation at rs1344706 on sleep neurophysiology and procedural memory consolidation in healthy participants.

**Methods/Design:**

Participants will be recruited from the Avon Longitudinal Study of Parents and Children (ALSPAC) on the basis of genotype at rs1344706 (*n* = 24). Each participant will be asked to take part in two nights of in-depth sleep monitoring (polysomnography) allowing collection of neurophysiological sleep data in a manner not amenable to large-scale study. Sleep questionnaires will be used to assess general sleep quality and subjective sleep experience after each in-house recording. A motor sequencing task (MST) will be performed before and after the second night of polysomnography. In order to gather additional data about habitual sleep behaviour participants will be asked to wear a wrist worn activity monitor (actiwatch) and complete a sleep diary for two weeks.

**Discussion:**

This study will explore the biological function of *ZNF804A* genotype (rs1344706) in healthy volunteers by examining detailed features of sleep architecture and physiology in relation to motor learning. Using a RBG approach will enable us to collect precise and detailed phenotypic data whilst achieving an informative biological gradient. It would not be feasible to collect such data in the large sample sizes that would be required under a random sampling scheme. By dissecting the role of individual variants associated with schizophrenia in this way, we can begin to unravel the complex genetic mechanisms of psychiatric disorders and pave the way for future development of novel therapeutic approaches.

**Electronic supplementary material:**

The online version of this article (doi:10.1186/s12881-015-0244-4) contains supplementary material, which is available to authorized users.

## Background

Genome wide association studies (GWAS) and subsequent large-scale meta-analyses have been extremely successful in identifying large numbers of associations (>100) between common genetic variants and schizophrenia [1]. However, one limitation of this approach is that phenotypic precision is often sacrificed in favour of the larger sample sizes needed to override the power constraints that come from both the limitations to measurement and small genetic effects associated with common genetic variation. The reduction of complex traits such as schizophrenia to the binary classification of case/control in GWAS leaves much uncertainty around the exact role of many of the associated variants in the aetiology of disease.

In a GWAS of 479 patients with schizophrenia and 2937 controls (with replication of associated variants in a further 16,726 subjects) the strongest evidence for association was shown for rs1344706 (odds ratio [OR] 1.12, *p* = 1.95 × 10^−7^), a single nucleotide polymorphism (SNP) within the *ZNF804A* gene [2]. This finding has since been replicated in a number of other GWAS [1, 3–5] including a fine-mapping study that failed to detect any genetic variants within the *ZNF804A* locus that were more strongly associated with schizophrenia than rs1344706, which in this analysis had an OR for schizophrenia of 1.10 [1.07 – 1.14] [6].

Since its discovery, rs1344706 has been linked to a number of biological phenotypes [7]. The variant has been shown to correlate with altered neuroanatomy [8–11], abnormal neurophysiology [12, 13] and in particular changes in neurocognitive phenotypes [11, 14]. For example, researchers using functional magnetic imaging (fMRI) found evidence that healthy subjects carrying the rs1344706 risk variant showed changes in functional connectivity of the right dorsolateral prefrontal cortex during a working memory task [15, 16]. Whilst cognitive defects are an established and well-studied feature of schizophrenia [17, 18], more recently, links have been made between the cognitive symptom dimension and another feature of the condition - abnormal sleep patterns. Sleep disturbances have long been described in schizophrenia [19, 20] with studies consistently reporting altered sleep architecture where patients show increased sleep latency (time taken to fall asleep), decreased total sleep time and other altered sleep variables - both on neuroleptic treatment and in the absence of medication [21, 22].

Sleep itself is a highly complex process that is tightly controlled by circadian rhythms and homeostatic mechanisms [23]. Sleep is known to be important for memory consolidation in both animals and humans [24, 25] with studies having shown improvements in both procedural and declarative memory consolidation following sleep [26]. Whilst even short naps lasting minutes to hours can result in better task performance compared to performance after an equally long period of wake time, longer periods of sleep lead to continued memory consolidation [27–29]. Moreover, sleep deprivation can severely impair memory consolidation [30]. The importance of sleep for memory consolidation in healthy participants is corroborated by impaired sleep-dependent memory consolidation in schizophrenia patients [31].

Human sleep can be classified into stages based on key features observed when monitoring brain activity (Electroencephalography; EEG), eye movement (Electrooculography; EOG) and muscle tone (Electromyography; EMG). The two main sleep stages are rapid eye movement (REM) sleep and non-rapid eye movement (NREM) sleep (further divided into stages 1–3) [32, 33]. In particular NREM sleep has been linked to memory consolidation in healthy volunteers [30]. More specifically, memory consolidation has been shown to correlate with spindle activity [34–36]. Spindle oscillations (a train of distinctive waves; 11–16 Hz, ≥0.5 s) are a feature found in the EEG during lighter NREM (stage 2) and arise from neuronal network oscillations spanning the cortex and thalamus. Spindle activity has also been shown to be reduced in patients with schizophrenia [37], and reduced spindle number and density correlated with impaired overnight improvement in a motor sequence task (MST) [38] (also in patients). Overall there is growing evidence that circuit abnormalities in schizophrenia link to changes in sleep physiology which in turn may contribute to the cognitive symptoms of schizophrenia [39, 40].

To the best of our knowledge the potential impact of variation in *ZNF804A* on sleep neurophysiology in patients with schizophrenia has not been characterised. The study of sleep phenotypes in patients with schizophrenia can be challenging, both in terms of recruitment of participants and study implementation. In many instances, issues around ethics, safety, and illness limit recruitment and bias samples to those with relatively mild disease severity. Further, results may be confounded by factors that are difficult to control, for example, medication. Where associations are observed, there are also issues around establishing the direction of causality. So far, efforts have been made to minimise limitations by recruiting medication-naïve participants [22] and by studying phenotypes in the first degree relatives of patients with schizophrenia [41]. We consider the value of an approach whereby the genetic control of phenotypes relevant to disease is investigated in healthy people, avoiding the aforementioned difficulties of working with patient groups.

Here we present a protocol designed to evaluate the efficacy of a recall-by-genotype (RBG) study design in investigating the impact of *ZNF804A* genotype (rs1344706) on sleep neurophysiology and memory consolidation in healthy subjects. In RBG studies, a sub-set of participants from an existing study are recruited on the basis of measured genotypic variation; existing biosamples can then be analysed or new data collected. This study has the potential to improve our understanding of both the genetic and neurophysiological basis of sleep and the biological basis for the association of *ZNF804A* variants with schizophrenia. In the longer term, linking sleep disturbances to cognitive deficits in psychiatric disorders could open up new avenues for the treatment of cognitive symptoms. Restoring normal sleep patterns in patients with psychiatric disorders may improve cognition, well-being and overall patient outcome [21].

## Methods/Design

### Study design

A RBG design will be implemented in a population-based cohort. Participants will be recruited based on their genotype at rs1344706 and extended sleep-wake and cognitive testing datasets will be collected to allow detailed characterization of phenotypes.

### Ethical considerations and informed consent

Ethical approval for the study has been obtained from the ALSPAC Ethics and Law Committee (ref. 9224). The data collection protocol has also previously been approved by The University of Bristol Faculty of Science Human Research Ethics Committee as part of a pilot study led by MWJ (ref. 8089). All participants will receive a participant information sheet prior to being recruited to the study and will be given the opportunity to ask questions both at the telephone screening and during the study nights. All participants will be asked to complete a written consent form on their first visit and they will be asked for continued consent verbally on their second visit. Participants will be free to withdraw from the study at any time. Participants will be appropriately reimbursed for their time and effort (as judged against other contemporary study initiatives) and all travel expenses will be reimbursed.

### Participant recruitment

Male participants of European ancestry aged 21–23 years will be recruited from the Avon Longitudinal Study of Parents and Children (ALSPAC) on the basis of homozygous status at rs1344706. Homozygotes will be identified using existing genome-wide data, as imputed to the 1000 genomes reference panel (Phase 1, Version 3) (for more details on the genotyping and imputation of the cohort, see Additional file 1). ALSPAC is a trans-generational prospective birth cohort that began with the recruitment of 14,541 pregnant women resident in Avon, UK with expected dates of delivery 1st April 1991 to 31st December 1992. Since then, the health and development of mothers and their children has been followed across the life-course [42] (for more details about the cohort, see Additional file 1). The target sample size for this study will be 24, made up of 12 minor (CC) and 12 major (AA) homozygotes at rs1344706; in the event that the genotype groups are not equal, recruitment will continue until there is a minimum of 12 participants in each. Researchers will remain blind to participant genotype throughout the recruitment and data collection phases of the study.

**Fig. 1:**
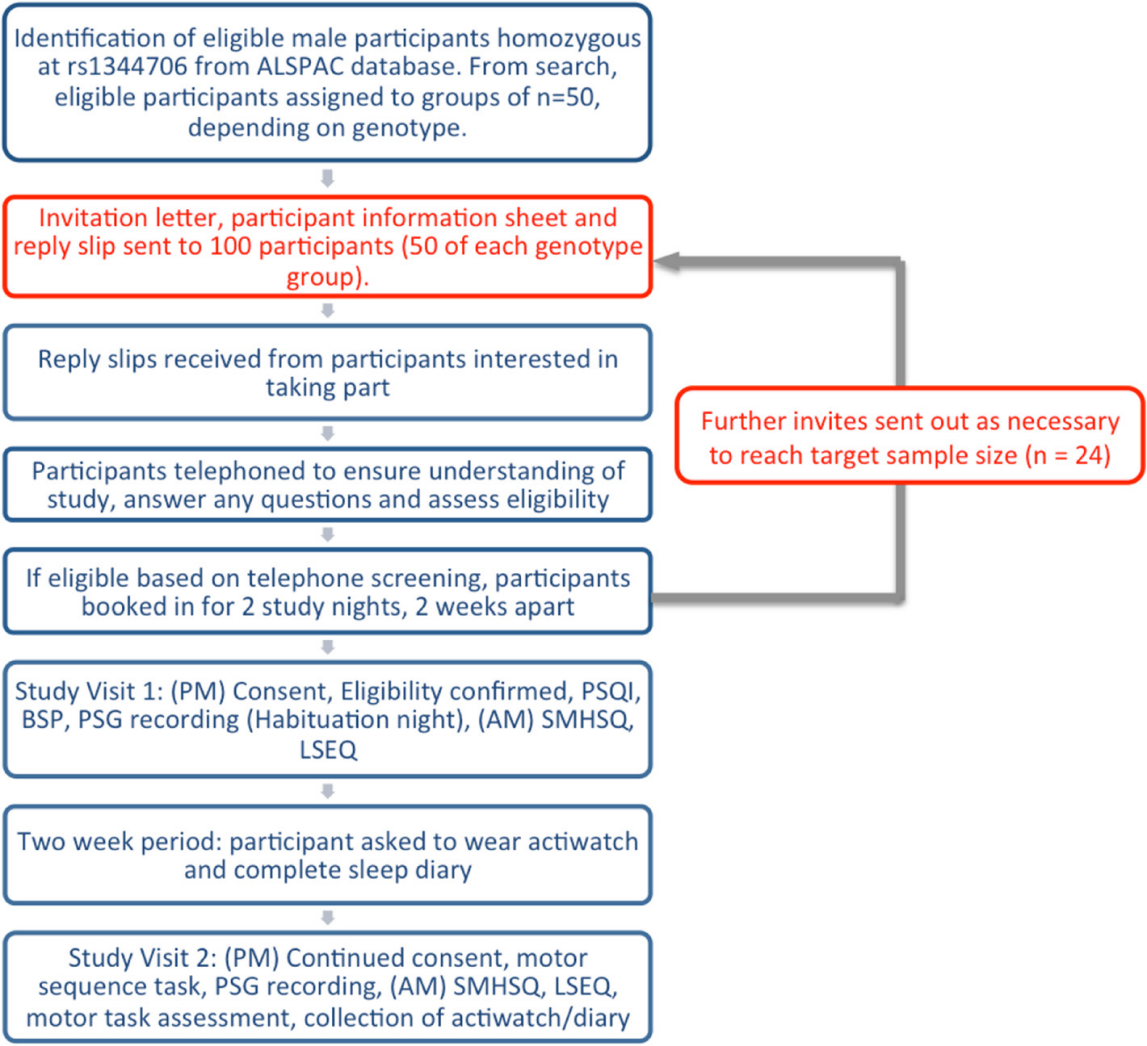
Study Workflow. Tasks in red were carried out by ALSPAC staff (not researchers) in order that researchers remained blind to participant genotype (ALSPAC – Avon Longitudinal Study of Parents and Children; BSP – Bristol Sleep Profile; LSEQ - Leeds Sleep evaluation Questionnaire; PSG – polysomnography; PSQI –Pittsburgh Sleep Quality Index; SMH - St Mary’s Hospital Sleep Questionnaire)

Invitations will be sent to selected ALSPAC participants, together with a participant information sheet and reply slip, in batches of 100, with an even split between the two genotype groups until the target sample size is reached. Invitations will be sent preferentially to ALSPAC participants for whom other relevant phenotypic data is already available; these include WISC (Wechsler Intelligence Scale for Children) results, n-back task data and PLIKS (Psychosis-like symptoms) questionnaire data. An overview of the participant recruitment process is shown in Fig. 1. All participants who volunteer to take part will undergo a telephone screening to check eligibility against the following criteria:

#### Inclusion criteria

- In good physical or mental health;
- Non-smoker;
- Able to give informed consent as judged by the investigator;
- Available to attend two overnight clinic visits.

#### Exclusion criteria

- Previously diagnosed with a sleep disorder;
- Substantive current or past medical history;
- Taking medications that may affect or induce sleep;
- Working a regular night shift (or has done within the last six months);
- Current substance abuse, including alcohol excess (more than 24 units a week).

Participants will be asked to refrain from drinking alcohol in the 24 h prior to attending the clinic. They will also be advised not to consume any caffeine-containing drinks after 4 pm on the day of their visit to the clinic. Participants will be asked a series of screening questions at each visit to verify their adherence to these instructions and to confirm their on-going eligibility for the study.

### Data collection

The data collection phase for each participant will be two weeks long, beginning and ending with a night spent in the sleep suite at the clinic during which a polysomnography (PSG) will be performed.

#### Actigraphy and sleep diary

During their first visit to the clinic, the participants will be issued with a wrist worn activity monitor (MotionWatch 8, CamNtech, UK), which they will be asked to wear for the two weeks between overnight sleep monitoring nights. The MotionWatch contains a miniature accelerometer to allow measurement and recording of physical movement of the wrist, which provides a close correlation to whole body movement [43]. These data are stored into an internal memory and then downloaded for analysis at the end of the study period and analysed using MotionWare software [43]. Participants will also be asked to record information about their bedtime, wake/rise times, naps, daytime activities and caffeine and alcohol intake in a standardised diary for the duration of actigraphic monitoring. This will provide detailed information about the participant’s habitual sleep behaviour with the diary information aiding interpretation of the actigraphy data. Both the MotionWatch and diary will be returned by the participant when they attend the clinic for the second time.

#### Questionnaires

Further behavioural data will be collected from each participant using a series of questionnaires. The Pittsburgh Sleep Quality Index (PSQI) [44] and Bristol Sleep Profile (BSP) [45] will be used at the first visit to assess the self-rated sleep quality and to indicate any specific sleep disturbance, respectively. Each participant will also be asked to complete the Edinburgh Handedness Inventory [46] which will be used to ascertain participant handedness ahead of the MST (see below). After each PSG recording, participants will be asked to complete the St Mary’s Hospital Sleep Questionnaire (SMH) [47] and the Leeds Sleep Evaluation Questionnaire (LSEQ) [48]; to collect information about subjective experience of their night in the sleep laboratory, as compared to a normal night.

#### Polysomnography

Each participant will stay in the sleep suite for two nights, one habituation night and one test night. A standard in-laboratory, overnight polysomnography (PSG), including video and audio recording, will be performed using Embla® sleep diagnostics equipment and RemLogic™ software (Natus Medical Inc., California). Electrodes will be placed according to the internationally standardised 10–20 system and data acquired using a standard PSG recording montage. Additional electrodes will be placed to monitor eye movements, submental muscle activity and heart rate throughout the recording. Following electrode placement and bio-calibration, the participant will be able to follow their usual evening routine and will be encouraged to go to bed at their usual bedtime. The participant will be woken as close as possible to their usual wake time and the sensors removed. Following the completion of the data collection phase of the study, PSG data will be manually scored by an experienced expert, blinded to participant genotype, based on standardised criteria [32, 33]. A range of sleep variables will then be derived from the annotated PSG recordings (see Table 1).

**Table 1:** A summary of data collected and corresponding outcome measures

	Data source	Outcome measures
Primary outcome measures	Polysomnography	Sleep variables derived from scored recordings, e.g. total sleep time (TST), sleep onset latency (SOL), sleep efficiency (SE), wake after sleep onset (WASO)
		Time spent in each sleep stage; S1, S2, Slow wave sleep (SWS) and rapid eye movement sleep (REM)
		Spectral composition of EEG data (delta, theta, alpha, sigma and beta frequency bands)
	Motor sequence task	Number of correct sequences pre- and post-sleep
		Percentage improvement
		Reaction time pre- and post-sleep
Secondary outcome measures	Actigraphy	Sleep/Wake estimates e.g. sleep period, sleep efficiency, total sleep time.
		Non-parametric circadian rhythm analysis (NPCA) e.g. 10 most active hours (M10), 5 least active hours (L5)
		Daily activity levels
	Sleep diary	Detailed information about rest/activity routine (to aid interpretation of actigraphy)
	Pittsburgh Sleep Quality Index	Sleep quality (over previous month)
	Bristol Sleep Profile	Assessment for sleep disorders (eligibility)
	St Mary’s Hospital Sleep Questionnaire	Sleep quality, latency, continuity, satisfaction etc. (study nights)
	Leeds Sleep Evaluation Questionnaire	Total Score and individual components; Getting to sleep, quality of sleep, awake following sleep, behaviours following awakening (study nights)
	Stanford Sleepiness Scale	Assessment of alertness prior to MST

#### Motor sequence task

On their second visit participants will perform a MST [38] approximately two hours before they go to bed and again the following morning. Prior to completing the MST the participant will be asked to complete the Stanford Sleepiness Scale [49]. During the task participants will be shown a sequence of numbers consisting of 5 elements (4-1-2-3-4) on a computer. They will be asked to type out this sequence with their non-dominant hand as fast and as accurately as possible for 30 s. Following this they will be given a 30 s break. This will be repeated 12 times.

### Data analysis

A range of primary and secondary outcome measures will be derived from the raw data, as outlined in Table 1. We plan to examine the difference in primary outcome measures across the recall strata, including a range of sleep variables and performance in the MST task.

## Discussion

Here we describe a study to investigate the impact of sequence variation in *ZNF804A* on sleep neurophysiology and memory consolidation in healthy subjects. Existing research shows that the rs1344706 variant in *ZNF804A* is associated with schizophrenia and a range of neuroanatomical and neurocognitive phenotypes. It is also known that in patients with schizophrenia, abnormal sleep phenotypes are associated with poorer memory consolidation during sleep. Building on this knowledge, this study aims to elucidate the aetiology of the association of rs1344706 with schizophrenia, in particular, whether the variant influences sleep neurophysiology and has a corresponding effect on memory consolidation.

The use of a RBG study design provides important advantages for this type of research question. It allows for more targeted selection of participants and as a result increases the efficiency of research and focuses the distribution of resources [50]. Participants, whose genotype has previously been determined, can be selected on the basis of having a certain genetic variant that is thought to contribute to a specific functional change. As a result fewer participants need to be recruited to achieve equivalent power to a similar population based study. In this study we will aim to recruit 24 participants, 12 in each homozygote genotype group. In comparison, based on a minor homozygote (CC) frequency for rs1344706 of 17 % in the ALSPAC cohort, approximately 70 individuals would need to be recruited and genotyped to ensure a similar number of participants in the minor homozygote group. This amounts to an approximate tripling of the study time required under the RBG model if a random sampling approach was used (from ~480 h to ~1400 h).

This property of RBG is particularly important when the data collection procedure is particularly labour and/or time intensive as in this study. In this sense, a RBG design represents the antithesis of GWAS with sample size able to be sacrificed in favour of phenotypic precision, with the guaranteed biological gradient providing optimal statistical power. We also expect rs1344706 to show larger effects as a result of using indices of neurophysiological function rather than exclusively cognitive measures [14]. Furthermore, the RBG approach builds on the theoretical developments around causal inference derived from Mendelian randomization and as such avoids the issues of confounding and reverse causality that commonly effect traditional observational studies [51].

Whilst the use of a RBG study design provides a new and powerful approach to research into the effects of genetic variation on phenotypes, it also creates new ethical challenges that should be considered [52]. It is important to provide information to participants about why they have been invited to the study but at the same time not to disclose genetic information, especially when the clinical consequences of this information are unclear or unknown. When this study was conceived careful consideration was given to its ethical implications. Both the ALSPAC Ethics and Law Committee and the ALSPAC Original Cohort Advisory Panel (OCAP) were consulted on decisions regarding recruitment and the disclosure of information to participants. In addition impartial information will be collected on the ethical issues surrounding the study. Whilst no individual genetic information will be disclosed, participants will be informed that they were recruited on the basis of their genotype and they will be given opportunities to ask questions about the study and RBG recruitment method. Researchers will remain blind to participant genotype throughout the data collection phase of the study, with genotype information only being released following the anonymization of the study data.

In summary, a RBG study is described that will contribute to our understanding of the aetiology of variation in sleep neurophysiology and the impact of genetic variants on this. By exploiting the analytical gains afforded through extremely precise phenotyping over informative genetic strata, this approach can simultaneously be financially viable (due to relatively small sample sizes) and analytically efficient. This study will help to further understanding of the neurophysiology of sleep and sleep disturbances. Whilst the sleep phenotypes studied may not necessarily be linked directly to disease, they could nevertheless provide information on pre-morbid phenotypes and improve understanding of disease pathophysiology. Following the completion of this pilot study, the results will be used to inform the planning of further larger studies, which may also look at other genetic variants associated with schizophrenia.

## Additional file

Additional file 1:
**The Avon Longitudinal Study of Parents and Children.** This file contains a description of the cohort used for the study, including details of participant recruitment and genotyping and imputation procedures. (PDF 72 kb)
